# Nurse-led safer opioid supply and HIV pre-exposure prophylaxis: a novel pilot project

**DOI:** 10.1177/20499361221091418

**Published:** 2022-04-17

**Authors:** Marlene Haines, Patrick O’Byrne

**Affiliations:** School of Nursing, University of Ottawa, Guindon Hall RGN 3051, 451 Smyth Road, Ottawa, ON K1H 8M5, Canada; School of Nursing, University of Ottawa, Ottawa, ON, Canada

**Keywords:** drug use, harm reduction, HIV pre-exposure prophylaxis, overdose crisis, safer opioid supply

## Abstract

**Introduction:**

HIV pre-exposure prophylaxis (PrEP) is an effective intervention for preventing HIV infections yet is largely unknown to and underutilized among people who use drugs.

**Methods:**

To better provide services to this group, we present a prospective, single-group interventional study involving the creation of a partnership between a safer opioid supply program and an HIV PrEP program, both of which were nurse-led.

**Results:**

Overall, HIV PrEP was offered to 42 individuals within the safer opioid supply program, resulting in 55% (*n* = 23) acceptance. Almost half of the group that accepted PrEP identified as female, and nearly all participants were homeless and did not have a primary care provider. While it was challenging to obtain routine PrEP follow-up labs per guideline recommendations due to poor venous access, most participants were able to successfully stay on PrEP and maintained good medication adherence. There were no PrEP discontinuations due to renal impairment and no participants tested positive for HIV.

**Conclusion:**

This novel integration of programs appeared to be a highly effective way to expand access to HIV prevention among people who use drugs. Given the historical and current mistreatment of people who use drugs within the healthcare system, rapport and trust were essential to the uptake of HIV PrEP services. Further, the importance of infectious disease screening among people who use drugs is underscored, and built-in program flexibility and low barrier access is essential.

## Introduction

HIV pre-exposure prophylaxis (PrEP) involves HIV-negative persons taking antiretroviral medications for prevention – not treatment – purposes, and the available literature shows that this intervention is upward of 99% effective when taken as prescribed.^1^ Real-world epidemiologic data show corresponding decreases in HIV incidence in cities with high rates of PrEP coverage among the persons most likely to be affected by HIV.^2^ In Canada and the United States, the groups with the highest HIV incidence and prevalence include gay, bisexual, and other men who have sex with men (gbMSM), trans persons, individuals of African, Black, or Caribbean (ACB) ethnicities, members of Indigenous communities, and people who use drugs (PWUD).^3^

While the literature contains many examples of PrEP use among gbMSM and trans populations, it is limited involving ACB, Indigenous, and PWUD populations. PWUD face considerable inequities in accessing healthcare, frequently reporting experiences of stigma, marginalization, and stereotyping.^4–9^ This inequitable access is also reflected in the context of infectious diseases – despite PWUD being considered a high-risk population for HIV acquisition, less than 15% are aware of what PrEP is, and less than 1% use PrEP.^3^ To address this situation, we expanded our nurse-led PrEP clinic (entitled PrEP-RN, for PrEP – Registered Nurse) to include PWUD who were enrolled in a downtown safer opioid supply (SOS) program. SOS is a public health initiative which seeks to address the social and health concerns related to the current toxic illicit drug supply.^10–14^ In 2020, approximately 17 individuals in Canada died each day from opioid toxicity – over 6000 people over the course of 12 months – representing the deadliest year on record. It is important to note that these numbers are likely much higher than this, given inconsistencies in reporting techniques across the country (including some regions not reporting at all).^15^

SOS exists to decrease harms related to substance use, such as the risk of overdose, participation in criminal activities, and associated trauma/mental health concerns through the prescription of pharmaceutical opioids.^16–18^ SOS often includes the prescription of a long-acting opioid for withdrawal (such as methadone, slow-release oral morphine, buprenorphine/naloxone, etc.) along with short-acting hydromorphone for cravings.^19^ This SOS program exists within a 24/7 supervised consumption site, and was designed with as few barriers as possible, such as flexible eligibility requirements, unobserved dosing, extended hours of operation, accessible outreach services, as well as being embedded within a homeless shelter and supervised consumption site. The SOS program is driven by individualized client goals and seeks to improve health through low barrier access to integrated health, social, and other services.

In this article, we describe our PrEP-RN SOS participants and highlight that this expansion was a novel way to include PWUD who were at high risk for HIV, but otherwise had limited access to PrEP.

## Methods

### PrEP-RN overview

As detailed elsewhere,^20–22^ PrEP-RN was a two-part nurse-led PrEP program, which comprised the following: (1) active-offer PrEP referrals by public health nurses and (2) a PrEP clinic within our sexually transmitted infection (STI) clinic. While anyone in our city could request and obtain PrEP through primary care or other PrEP clinics, PrEP-RN was designed to facilitate rapid PrEP initiation for persons at elevated risk for HIV acquisition.

Active-offer differs from traditional referral systems in that nurses approach clients with objective risk factors to offer them PrEP. To explain further, in our jurisdiction, public health nurses receive reports about persons who were newly diagnosed with STIs/HIV. As part of PrEP-RN, our nurses discussed PrEP with every person who had any of the following reportable infections: rectal gonorrhea or chlamydia or a new diagnosis of infectious syphilis (this refers to new syphilis infections, less than 12 months, which can be transmitted to others). These nurses also discussed PrEP with anyone who was named as a contact of a person newly diagnosed with HIV. Any patient who agreed to PrEP was offered an appointment at the PrEP-RN clinic or any other local clinic that offered PrEP. Those who accepted PrEP-RN were booked to see a PrEP-RN nurse, whereas a referral was completed when patients selected a different local PrEP clinic. We additionally offered PrEP-RN appointments to any patient to whom we prescribed HIV post-exposure prophylaxis (PEP) or who, based on nursing clinical judgment, was deemed high risk for HIV acquisition despite not fulfilling foregoing risk criteria (e.g. an individual who participates in survival sex work and regularly engages with new sexual partners). Per our previous research, from 5 August 2018 to 4 March 2020, we referred 347 persons for PrEP-RN, of whom 47% accepted PrEP. These participants were 100% male, 99% gbMSM, and 54% white, with 46% reporting an income >$50,000CAD and 47% reporting having a primary care provider.^20–23^

PrEP-RN followed the Canadian guidelines on PrEP management,^1^ using emtricitabine-tenofovir disoproxil fumarate fixed tablet medication, with clinical assessments for PrEP indication and HIV seroconversion symptoms, plus baseline testing for HIV, renal function, hepatitis A, B, and C, and other STIs. Repeat clinical and serologic assessments occurred after 1 month of PrEP use, then every 3 months. STIs were managed according to current guidelines, with referrals being sent for hepatitis management.

The SOS PrEP-RN study is a prospective, single-group interventional study. Current SOS clients were directly approached for recruitment by frontline nurses working within the SOS program. Both the research project and an overview of PrEP were provided to all SOS clients. Should the client wish to participate, the SOS nurse immediately initiated the intake process for PrEP. Indeed, intake assessments, bloodwork, and counseling were performed by an SOS nurse who was trained on PrEP, and who completed standardized PrEP intake and assessment forms. All intake forms and laboratory values were reviewed by the core PrEP-RN team, with prescriptions for PrEP sent when indicated to the pharmacy which dispensed the SOS medications. SOS PrEP-RN participants thus obtained their PrEP medication daily when they presented for SOS medication administration. This enabled daily tracking of medication use. Notably, SOS care delivery and medications were publicly funded and provided in spaces that were geographically close to where PWUD reside, and which operated with expanded hours (16 h per day, 7 days per week) without scheduled appointments to maximize the ability for PWUD to obtain their SOS and PrEP medications.

### Data collection and analysis

We collected data in two ways. First, we maintained a record of all persons to whom we offered PrEP-RN, with reasons for declining being noted verbatim. Second, we manually extracted data from participants’ charts and recorded them in an Excel file. The variables we extracted included the following: age; sex; ethnicity; language; primary care attachment; housing status; hepatitis A, B, and C status; STI/HIV screening results; and medication use. We analyzed these data using descriptive statistics for rates, frequencies, and averages.

## Results

Over the first 6 months of SOS PrEP-RN (5 December 2020 to 12 June 2021), we offered 42 persons PrEP: 55% (*n* = 23) accepted and completed the intake to start PrEP, 24% (*n* = 10) declined, and 21% (*n* = 9) were ineligible; another four participants discontinued SOS before a PrEP offer could be made. Among the 10 persons who declined, 5 felt they had no risk for HIV and 5 wished to follow up with their primary care provider. Among the nine who were ineligible, seven were already diagnosed with HIV and two were unable to accept PrEP due to mental health issues at the time.

Among the 23 participants who accepted PrEP, the average age was 36 years (range: 24–60). Of these participants, 43% (*n* = 10) identified as female and 57% (*n* = 13) as male, 70% (*n* = 16) were White and 22% (*n* = 5) were members of Indigenous communities, 26% (*n* = 6) identified as Francophone Canadians, 96% (*n* = 22) reported being homeless, and 78% (*n* = 18) denied having a primary care provider. Of these 23 participants, 17% (*n* = 4) never initiated PrEP, 65% (*n* = 15) continued it, and 17% (*n* = 4) discontinued. Overall, 36% (*n* = 15/42) of the total group to whom we offered PrEP remained engaged with this intervention for the duration of the study. Among the 15 participants who remained engaged in PrEP-RN, 8 identified as female, and 9 were White, 5 were Indigenous, and 14 had hepatitis C antibodies, with 10 having positive RNA; one person was diagnosed with gonorrhea and subsequently treated. Notably, 8 of the 10 participants with positive hepatitis C RNA results were started on hepatitis C treatment following their diagnosis.

Among the 11 participants who reached PrEP follow-ups at 1, 3, and 6 months, 6 were off-schedule and 5 were on-schedule for the scheduled follow-ups, meaning that most required medication extensions of 1–2 weeks. These extensions often occurred because participants were unable to stay for serology, engaging in the program at times when labs were closed and bloodwork could not be delivered in time (e.g. evenings and overnight), and, most commonly, difficult venous access. Indeed, return visits to reattempt serology were often required. Notably, however, no participant discontinued PrEP due to impaired renal function due to PrEP use, and no one tested positive for HIV. Regarding adherence, the Canadian PrEP guidelines indicate that PrEP may be less effective if a person misses greater than or equal to three consecutive doses. From our sample, 80% of the time participants missed fewer than three consecutive doses, with 55% of participants never reaching this threshold number of missed doses.

## Discussion

In this article, we reported on the PrEP-RN expansion to include persons enrolled in an SOS program in downtown Ottawa, Canada. Over 6 months, PrEP was accepted by over half of those to whom we offered PrEP, with about one-third remaining on PrEP at 6 months. Notably, nearly half of the SOS PrEP participants were female, nearly all were homeless, all used drugs, over three-quarters did not have a primary care provider, and about one-quarter identified as members of Indigenous communities. While our study period was short and the sample size was small, our results raise a few important points for consideration.

First, our results are a proof-of-concept that SOS programs are an effective way to expand PrEP delivery to a larger pool of persons who are at high risk for HIV acquisition. While current strategies have demonstrated strong uptake among gbMSM, such usage has not been observed in other groups with elevated HIV risk. Novel approaches are thus required to engage PWUD, ACB, and Indigenous persons. Comparing the larger PrEP-RN cohort with the SOS program, we increased enrollment among females (from 0% to 43%), individuals who are homeless (from 0% to 96%), and those without primary care attachment (from 38% to 78%). Taken as a whole, these data suggest that SOS programs should consider offering PrEP independently or should – as we did – create partnerships between SOS and PrEP clinics to maximize the skills and resources of each set of clinicians and decrease barriers to access to marginalized individuals at high risk for HIV acquisition. Indeed, by integrating PrEP into care that is directly related to the lives of PWUD, including establishing linkages to SOS prescribers and a pharmacy that would dispense daily PrEP along with daily SOS medications, we helped overcome social determinants that impede the health of PWUD, including access to care, cost of medications, and the ability to take and use treatments. In this way, the merger of SOS and PrEP programs could address major health inequities and decrease both the sequelae of a toxic drug supply (including overdoses) and ongoing HIV transmission.

Second, while low-barrier, flexible access to PrEP within the SOS program was essential to maintaining accessibility for PWUD, the established rapport and trust among the SOS nursing team and SOS participants likely played a large role in the success of this collaboration. As outlined at the outset of this article, PWUD often experience intense stigma, marginalization, and criminalization within the healthcare system, resulting in mistrust and difficulties in accessing appropriate and acceptable care. Clients engaged in SOS had already developed a therapeutic relationship with the SOS nursing staff, resulting in ease of discussions regarding PrEP initiation and follow-up. Further, the SOS nurses were able to bring care to the participants in the shelter and supervised consumption site, not only removing the barrier of physical distance with regard to attending a different clinic but also allowing for simplified, comprehensive care in a familiar environment.

Third, these results suggest the importance of hepatitis C screening among PWUD. While this is not a new finding (due to the well-known elevated prevalence of hepatitis C among this population), what our findings add are the utility of using this infection as an objective indicator for potential HIV acquisition. While bacterial STIs (gonorrhea, chlamydia, and syphilis) are established risk factors for HIV acquisition among gbMSM, we only identified 1 such infection in this cohort, although we identified 10 active hepatitis C infections. This contrasts with the zero HIV infections we observed, which is likely due to artifact of a small study sample but also may be due to high PrEP adherence and efficacy enabled by a daily combination of PrEP and SOS medication dispensing accessible within a low-barrier environment (e.g. 24/7 supervised consumption site).

Fourth, our results highlight that any benefits that could materialize by providing PrEP to PWUD will only materialize if clinicians accept that these patients may require some added flexibility in follow-up timelines. While 80% of the time participants achieved near-perfect adherence, extensions of 1–2 weeks over the routine follow-up periods at 1 and then every 3 months were routinely required. This does not mean that recommended healthcare practices should not be observed for PWUD, but rather, that there must be an acceptance that serologic follow-up 4 times per year can be difficult for these patients. Instead, accepting that delays may occur acknowledges the lived experiences and life contexts of PWUD and addresses the social inequities that surround their existence. Indeed, modified hybrid SOS and nurse-led PrEP clinics may be one way to successfully move forward in reducing barriers, inequities, and health sequelae. To our knowledge, this is the first known of such integration of programs.

## Conclusion

In this article, we presented the implementation data for the expansion of a nurse-led PrEP clinic to include PrEP to PWUD who receive opioid SS. Our findings support the continuation of this model of care and serve as a proof-of-concept for expansion, both locally and to other sites in Canada and abroad. Indeed, we found that, by delivering care in a novel way, we were able to more broadly expand PrEP access to groups at high risk for HIV. This article thus serves as some foundational evidence about how to provide PrEP delivery for PWUD particularly in the setting of health inequities. Likely, our program would not have worked had access to care and medication not been free, and had we not delivered care in locations that are amenable to PWUD. With these lessons and the initial success of our program, it is hoped that HIV incidence among PWUD could decrease, and it is moreover hoped that others will take up this approach to care delivery to expand it more broadly.
